# Collaboration and topic switches in science

**DOI:** 10.1038/s41598-024-51606-6

**Published:** 2024-01-13

**Authors:** Sara Venturini, Satyaki Sikdar, Francesco Rinaldi, Francesco Tudisco, Santo Fortunato

**Affiliations:** 1 Department of Mathematics “Tullio Levi-Civita”, University of Padova, 35121 Padua, Italy; 2 Luddy School of Informatics, Computing, and Engineering, Indiana University, Bloomington, IN 47408 USA; 3 School of Mathematics, The University of Edinburgh, Edinburgh, EH93FD UK; 4 School of Mathematics, Gran Sasso Science Institute, 67100 L’Aquila, Italy

**Keywords:** Physics, Statistical physics, thermodynamics and nonlinear dynamics, Complex networks

## Abstract

Collaboration is a key driver of science and innovation. Mainly motivated by the need to leverage different capacities and expertise to solve a scientific problem, collaboration is also an excellent source of information about the future behavior of scholars. In particular, it allows us to infer the likelihood that scientists choose future research directions via the intertwined mechanisms of selection and social influence. Here we thoroughly investigate the interplay between collaboration and topic switches. We find that the probability for a scholar to start working on a new topic increases with the number of previous collaborators, with a pattern showing that the effects of individual collaborators are not independent. The higher the productivity and the impact of authors, the more likely their coworkers will start working on new topics. The average number of coauthors per paper is also inversely related to the topic switch probability, suggesting a dilution of this effect as the number of collaborators increases.

## Introduction

Modern science has become increasingly collaborative over the past decades^1^. Large teams have become almost necessary to tackle complex problems in various disciplines, requiring a large pool of knowledge and skills. On the other hand, small teams may introduce novel paradigms^2^.

A powerful representation of the collaborative nature of science is given by a collaboration network, in which nodes are authors, and two nodes are connected if they have coauthored at least one paper. With the growing availability of bibliometric data, collaboration networks have been extensively studied, and their structural properties are now well known^3–6^. Collaboration networks are concrete manifestations of *homophily* between scholars, i.e*.*, of the tendency of individuals to interact with people similar to themselves. People working on the same topic or problem may decide to team up and leverage their respective skills to increase their chances of discovering new results. This is an example of *selection*, where homophily results from the choice of people to engage with similar individuals. On the other hand, collaboration could also induce *social influence*, in that scholars might affect the future behavior of their coauthors. For a thorough discussion on homophily, selection, and social influence, we refer the reader to chapter 4 of the book by Easley and Kleinberg^7^.

Coauthors often expose us to new tools, methods, and theories, even when the latter is not being used for the specific project carried out by the team. The link between diffusion of knowledge and collaboration has been highlighted and explored for some time. For instance, it is known that knowledge flow occurs with a greater probability between scholars who have collaborated in the past^8^ and those who are in close proximity in the network^9^.

In particular, once scholars discover new research topics, they may decide to work on them in the future. Switches between research interests have become increasingly frequent over time^10^ and have recently been subjected to investigation^11,12^. The decision to switch may actually be induced by the coauthors in a social contagion process^13–17^ where scholar *a*, who spreads the new topic, influences scholar *b* to adopt it. For this reason, epidemic models have been applied to describe the diffusion of ideas^18–20^. In these models, an *infected* individual *a* exposes a *susceptible* individual *b* to a disease with a certain probability of getting infected and continuing the spread. In the case of an idea or a topic, the infection spreads if *b* adopts the new idea or starts working on the new topic. On a macro level, dynamics within collaboration networks like topic switches guide the evolution of disciplines^21,22^.

Here we present an extensive empirical analysis of the relationship between topic switches of scientists and their collaboration patterns. We distinguish active authors, i.e*.*, those who have papers on the new topic, from inactive authors who have never published in that area. For simplicity, we focus only on the first-order neighborhoods in the collaboration network. We find that the probability that the inactive coauthors of an active scholar switch topic grows with the productivity and impact of the latter. The larger the average number of inactive coauthors of active scientists, the smaller the effect. Also, the topic-switch probability for an inactive scholar grows with the number of their active coauthors, with a profile suggesting that the contributions of each coauthor are not independent.

**Figure 1:**
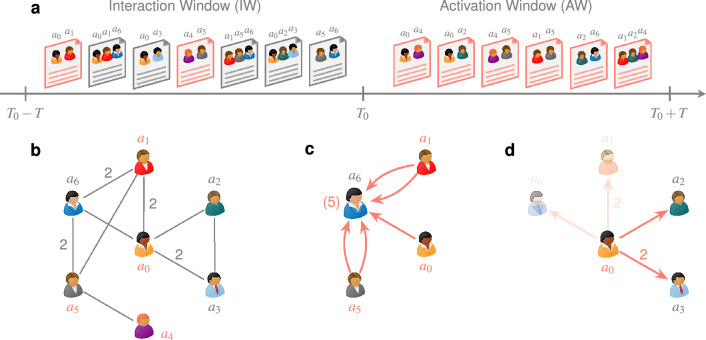
Schematic setup for our analysis. (**a**) Stream of papers across interaction (IW) and activation (AW) windows. Papers tagged with the focal topic *t* are marked in red. (**b**) Author collaboration graph at the end of IW. Authors $a_i$ and $a_j$ are linked by an edge of weight *k* if $a_i$ coauthored *k* papers with $a_j$ within the IW. The authors active in the focal topic by the end of IW are marked in red. (**c**) Focus: inactive authors. Inactive author $a_6$ has five active contacts from three sources {$a_0, a_1, a_5$} derived from the collaboration graph in (**b**). (**d**) Focus: active authors. Active author $a_0$ has four coauthors {$a_1, a_2, a_3, a_6$}, of whom $a_1$ is already active, and $a_6$ also collaborated with $a_1$ in the IW. This leaves the subset of exclusive inactive coauthors $\{a_2, a_3\}$. Within this subset, only $a_2$ becomes active in the AW, resulting in $a_0$’s source activation probability of $\tfrac{1}{2} = 50\%$. Additionally, $a_2$ writes their first paper with $a_0$ in the AW.

## Results

We use the scientific publication dataset OpenAlex^23^. We present the results for twenty topics belonging to three disciplines: Physics, Computer Science, and Biology & Medicine. See “Methods” for details.

Our approach is inspired by the pioneering work by Kossinets and Watts on social network evolution^24^. In it, the authors estimated *triadic closure* of two individuals *a* and *b*, i.e., the probability that *a* and *b* become acquainted as a function of the number of common friends. They took two snapshots of the network at consecutive time ranges: in the earlier snapshot, one keeps track of all pairs of disconnected people, and in the latter, one counts how many of those pairs become connected. A similar approach has been adopted to compute *membership closure*, i.e*.*, the probability that an individual starts participating in an activity having been connected to *k* others who participate in it^25^. We now describe how we adapt this framework to measure how collaborations induce topic switches.

Given a scientific topic *t*, reference year $$T_0$$, and window size *T*, we construct two consecutive non-overlapping time ranges spanning years $$[T_0-T, T_0)$$ and $$[T_0, T_0 + T)$$ respectively. We call the first range the *interaction window* (IW), where we track author interactions in the collaboration network, and the latter range, the *activation window* (AW), where we count topic switches. We then identify the set of *active* authors *A* who published papers *P* on topic *t* during the IW. For example, in Fig. 1a, $$A = \{a_0, a_1, a_4, a_5\}$$. We construct the collaboration network *G* by considering all papers $$P^\prime$$ written by authors $$a \in A$$ after *a* becomes active. Note that $$P^\prime$$ includes papers outside of *P*, like the ones drawn in gray in Fig. 1a. We classify the non-active authors in *G* as *inactive* authors who are the candidates for topic switches in the AW. They turn active when they publish their first paper on topic *t*. In Fig. 1b, authors $$a_2, a_3$$, and $$a_6$$ are inactive, with $$a_2$$ and $$a_6$$ becoming active in the AW. Furthermore, we rank each active author $$a \in A$$ based on two metrics of scientific prominence: *productivity* and *impact*, described in Methods, and calculated at the end of the IW to capture the current perception of *a*’s scholarly output. Finally, for each metric, we identify and mark the authors who rank in the top and the bottom 10%.

Given this general setup, we conduct two complementary experiments that we describe in depth in the following sections. In Experiment I, we measure membership closure among inactive authors to quantitatively assess how past collaborations with active authors manifest in topic switches. In Experiment II, we instead focus on active authors, quantifying the propensity of their inactive coauthors to start working on their topic of expertise. All the measures used in these sections are formally defined in Methods.

**Figure 2:**
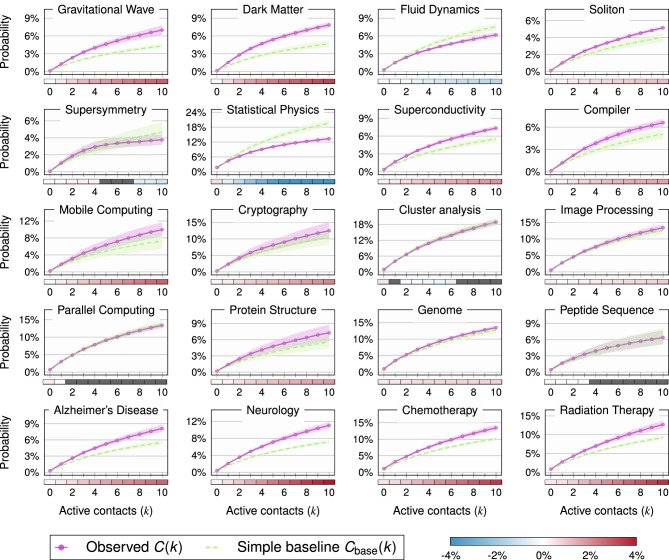
Experiment I. Cumulative target activation probability (in purple) for inactive authors in the AW with shaded 95% confidence intervals. For each *k*, the *y*-value indicates the fraction of inactive authors with at least *k* active contacts in the IW who became active in the AW. The dashed green line with shaded errors represents the baseline described in the text, corresponding to independent effects from the coauthors. The heatmap below the *x*-axis shows the mean difference between the observed and baseline curves for each *k* value. It is gray if the 95% confidence interval contains 0, denoting the *k*-values where the points are statistically indistinguishable at *p*-value 0.05. Positive and negative deviations from the baseline are in red and blue, respectively.

### Experiment I

Here we investigate membership closure among inactive authors. Specifically, we will answer the following questions:

- How is the probability of topic switches related to *k*, the number of contacts with active authors?
- Does this probability depend on the relative prominence of the active authors?

To compute the measure, we first must define what construes as contact with an active author in the IW. We consider two definitions as described below.

1. The number of active coauthors, with the same coauthor counted as many times as the number of collaborations. In the collaboration network, this corresponds to the weighted degree when considering only active coauthors.
2. The number of papers written with active coauthors.

For example, in Fig. 1c, author $a_6$ has five contacts based on the first definition (two each from $a_1$ and $a_5$ and one from $a_0$), and three if we use the second (the second, the fifth, and the seventh papers in the IW). We report the findings based on the first definition in the main text. The results from the second definition do not alter the main conclusions and can be found in Supplementary Figs. S1 and S2 online.

To address the first question, we compute the cumulative *target activation probability* *C*(*k*), i.e*.*, the fraction of inactive authors who become active in the AW as a function of the number of contacts *k*. In Fig. 2, we plot *C*(*k*) (in purple) for each of the twenty topics under investigation. Error bars derive from averaging over different time windows for each field. As expected, we see an increasing trend. In particular, the jump from *k* = 0 to *k* = 1 is remarkable, showing that the probability of *spontaneous* activation in the absence of previous contacts (*k* = 0) is much lower than that of activation through collaboration (*k* $\ge$ 1). We observe that the higher the number of contacts, the larger the probability. Most of the growth occurs for low values of *k*.

To put these numbers in context, we consider a *simple baseline* $C_\text {base}(k)$ where we assume each contact has a constant, independent probability of producing a topic switch. Within each topic, we compute the difference between the curves for each value of *k* (see “Methods”) over all reference years and plot them below the *x*-axis. Except for the topics of Cluster Analysis, Parallel Computing, and Peptide Sequence, the observed curves deviate from the baseline. This provides some empirical evidence to ascertain that the baseline cannot capture the nuances in the observed data. A positive deviation for the majority of the topics indicates a compounding effect. Fluid Dynamics and Statistical Physics are exceptions, as they undershoot the baseline. This may be because they are broad interdisciplinary fields unlike the others, and having collaborators in different fields may lessen their effect.

**Figure 3:**
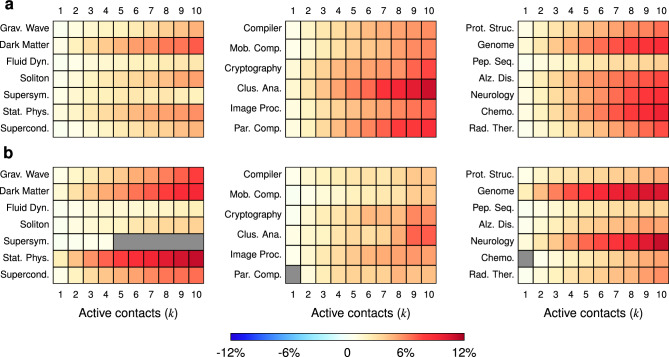
Heatmaps showing the mean difference between the cumulative target activation probabilities of the inactive authors in the AW who had exclusive contacts with the top 10% and bottom 10% of active authors, respectively, selected according to productivity (**a**) and impact (**b**) in the IW. The cells are gray if the 95% confidence interval contains 0. The topic names have been abbreviated to save space. The majority of red cells indicate that the cumulative target activation probabilities for contacts with the top 10% are higher than those with the bottom 10%.

Next, we explore the second research question, checking if the contact source’s prominence affects activation chances. Recall that in every IW for a topic, we select active authors in the top 10% and the bottom 10% based on productivity and impact. This separates the most prominent active authors from the least prominent. To mitigate confounding effects, we only consider the subset of inactive authors who are neighbors with strictly one of the two sets of active authors. In Fig. 3, we assess the significance of the difference between the cumulative target activation probabilities for inactive authors in contact with active authors in the two bins. Each heatmap row corresponds to a topic, and the color of each cell indicates whether the difference is positive (red), negative (blue), or non-significant (gray). The two panels correspond to prominent authors selected based on productivity (panel **a**) and impact (panel **b**). For productivity, all differences are significant and positive, meaning that contacts with highly productive active authors lead to higher target activation probabilities. For impact, there are a handful of exceptions. Overall, having prominent contacts increases the target activation probability.

### Experiment II

Here we focus on the active authors and their collaborators. For every active author *a*, we consider the subset of their inactive coauthors who have *exclusively* collaborated with *a* in the IW. We call this set the exclusive inactive coauthors of *a*. For example, in Fig. 1d, active author $a_0$ has four coauthors $\{a_1, a_2, a_3, a_6\}$, of whom only $a_2$ and $a_3$ exclusively collaborate with $a_0$ in the IW. We do this because effects due to active authors different from *a* would be difficult to disentangle and could confound the analysis and the conclusions. The relevant measure here is the *source activation probability* $P_s^a$, i.e*.*, the fraction of exclusive inactive coauthors who become active in the AW. The fraction controls for the collaboration neighborhood sizes which could vary widely for different scholars. In Fig. 1d, $P_s^a$ for $a_0$ is $\tfrac{1}{2}$ = 50%, as only $a_2$ becomes active in the AW.

**Figure 4:**
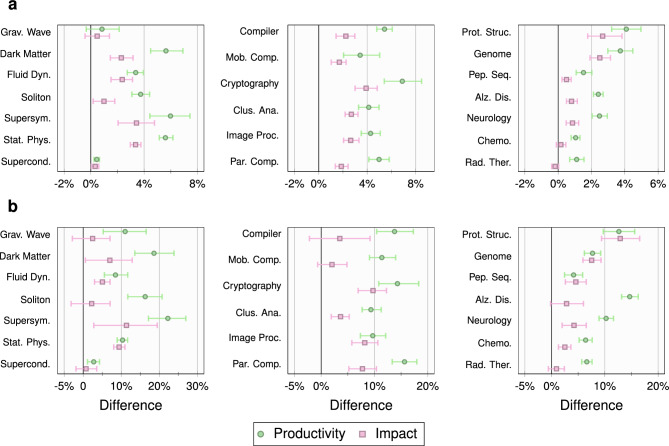
Experiment II. Results for $f^*= 0.10$. (**a**) The mean and 95% confidence interval of the means of the difference between the cumulative source activations of active authors in the top 10% and bottom 10% based on productivity (green circles) and impact (pink squares). (**b**) The mean and 95% confidence interval of the means of the difference between the chaperoning propensities of active authors in the top 10% and bottom 10% based on productivity (green circles) and impact (pink squares). The topic names have been abbreviated to save space. A positive difference indicates that the effect is stronger for the top 10% active authors.

For a given set of active authors, we obtain $C_s$, the *complementary cumulative probability distribution* of their source activation probabilities. We select the pools of the most and the least prominent authors as described in Experiment I. The relative effects of the two groups are estimated by comparing the *cumulative source activations*, i.e*.*, points on the respective cumulative distributions at a specific threshold $f^*$. Results are reported in Fig. 4a for a threshold $f^*= 0.10$. Our conclusions also hold when considering a threshold $f^*= 0.20$, which can be found in Supplementary Fig. S3 online.

In Fig. 4a, each row corresponds to a topic. The different ranges represent the $95\%$ confidence intervals of the mean difference between the cumulative source activations for the two pools of authors for productivity (green) and impact (pink), respectively. For productivity, the difference is significant for all topics but one (Gravitational Wave). The differences are somewhat less pronounced for impact, but are still significant in most cases.

To further corroborate this finding, we specialize the analysis by checking how many exclusive coauthors of *a* also published their first paper on topic *t* in the AW with *a*. This is a way to assess the *chaperoning propensity* of active authors^26^, and we define the measure in Methods. In Fig. 4b, we report the $95\%$ confidence intervals of the average difference between the chaperoning propensities for the most prominent and the least prominent active authors for threshold $f^*= 0.10$. Similar to Fig. 4a, we find that the more productive/impactful an active author is, the more likely their coauthors will start working with them on a new topic. Results for $f^*= 0.20$, which confirm this trend, can be found in Supplementary Fig. S4 online.

**Figure 5:**
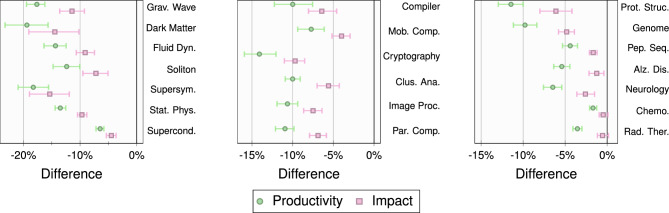
Dilution effect. Results for $f^*= 0.10$. The mean and 95% confidence interval of the mean of the difference between the cumulative source activations of active authors in the top 20% and bottom 20% bins, based on the average number of coauthors, among the top 10% active authors in productivity (green circles) and impact (pink squares). The topic names have been abbreviated to save space. A negative difference across the topics indicates a *dilution* effect, wherein coauthors of prominent active scholars with fewer collaborators (on average) are more likely to switch topics.

While our analysis clearly shows that prominence is a factor, one may wonder if the number of coauthors also plays a role. We posit that, on average, the more collaborators one has, the more tenuous the contact with any of them will be, resulting in lower source activation probabilities. From each group of most prominent authors, we, therefore, pick the top and the bottom 20% based on the average number of coauthors on papers published with exclusive inactive coauthors. By construction, this excludes any paper written on the focal topic. In Fig. 5, we perform the same analysis as in Fig. 4 for the two pools of authors described above. We observe that the confidence intervals of the differences lie to the *left* of zero, i.e*.*, are negative. For productivity, all values are significant. For impact, there are only two topics (Chemotherapy and Radiation Therapy) that are not significant. Overall, inactive coauthors of prominent authors with more collaborators have a lower probability of switching topics. This is consistent with the intuition that the interactions with each coauthor are less frequent/strong in that case and, consequently, less effective at inducing topic switches.

## Discussion

Collaboration allows scholars to deepen existing knowledge and be exposed to new ideas. In this paper, we assessed if and how collaboration patterns affect the probability of switching research topics. We determined that the probability for a scholar to start working on a new topic depends on earlier contacts with people already active in that topic. This effect is proportional to the number of contacts, with more contacts resulting in higher probabilities. In most topics, this behavior is distinct from a simple baseline assuming independent effects from the contacts, which likely indicates effects of non-dyadic interactions that prompt further investigation.

Similarly, we measured the probability that inactive coauthors of an active author end up publishing on the new topic, which singles out the effect of the association with that author in the activation process. Specifically, we checked whether the activation probability depends on some features of the active authors. We found that the more prolific and impactful authors have higher chances of inducing coauthors to switch topics and become coauthors in their first paper on the topic.

We stress that, by design, previous interactions between inactive and active authors are limited to works dealing with topics different from the focal topic. Therefore, our analysis suggests that an active author may expose an inactive one to a new topic, even when their interactions do not directly concern that topic. This underlines the social character of scientific interactions, where discussions may deviate from the context that mainly motivates them.

Furthermore, we showed that the larger the number of coauthors of an active author, the lower the chance of a topic switch. This is consistent with a *dilution* of the effect, resulting from the inability to interact strongly with collaborators when their number is large. To the best of our knowledge, we are disclosing this effect for the first time.

A possible explanation of our findings is that topic switches result from a social contagion process, much like the adoption of new products^15,27^, or the spreading of political propaganda^17^. However, we cannot discount selection effects in observational studies like ours^28^. Having large numbers of active coauthors on a topic may be associated with strong latent homophily between the authors, which may facilitate the future adoption of the topic even without interventions from the active authors. Therefore, the effects we observed may be due to a combination of social contagion and selection.

Our work uses OpenAlex, a valuable open-access bibliometric database. We rely on their author disambiguation and topic classification algorithms to conduct the analyses. These processes are inherently noisy and can introduce implicit biases. In addition, there appears to be incomplete citation coverage which might partly explain why the results for impact are not so robust as those for productivity. Future releases of OpenAlex might mitigate these problems. To counter these issues, we repeated our analysis on multiple topics from three distinct scientific disciplines. While the size of the effects varies with the topic, the paper’s main conclusions hold across topics, with very few exceptions.

In conclusion, our work offers a platform for further investigations on the mechanisms driving topic switches in science. A thorough understanding of these mechanisms requires effective integration of all factors that may play a role. Besides productivity and impact, topic switches may be affected by the institutional affiliations of those involved. On the one hand, it is plausible that people in the same institution have more chances to interact and affect each other’s behavior. On the other hand, collaborations with people from renowned institutions are expected to weigh more in the process. Another discriminating factor could be the number of citations to the collaborator’s papers. The higher the number of citations, the closer the association between collaborators. We could also include the scientific affinity between coauthors through the similarity of their papers. Modern neural language models^29,30^ allow to embed papers and, consequently, authors in high-dimensional vector spaces, where the distance between two authors is a good proxy of the similarity of their outputs. The analysis we have conducted here can be extended to other sectors of human activity where collaboration plays a key role, like software development and patent design.

## Methods

Data We analyze papers from the February 2023 snapshot of the bibliometric dataset OpenAlex: the successor to Microsoft Academic Graph (MAG). We found incomplete citation coverage for papers published before 1990. So, we only consider papers published between 1990 and 2022 and having at most thirty authors. Papers are tagged with *concepts* (topics) by a classifier trained on the MAG. We use concept tags to construct snapshots for three fields: Physics, Computer Science (CS), and Biology and Medicine (BioMed). Physics contains 19.7M papers, while CS and BioMed each have 27.6M and 43.52M papers, respectively. Within each domain, we select seven, six, and seven topics, respectively.

Within each topic, we consider reference years between 1995 and 2018, where the respective interaction and activation windows contain at least 3000 papers. This threshold ensures a critical mass of papers and authors to conduct the analyses. Each topic we selected has at least ten reference years satisfying the constraint. The statistical tests in the manuscript are aggregated over the different reference years. More information is available in Supplementary Tables S1–3 online.

Overlap coefficient We use the overlap coefficient to measure the degree of overlap between the different sets of authors picked based on productivity and impact.

$$\begin{aligned} \text {Overlap}(A, B) = \frac{|A \cap B|}{\min (|A|, |B|)} \end{aligned}$$

In our case, the two sets are the same size, so a score of 10% implies that both sets share 10% of the elements.

Author ranking metrics Let *P* be the set of papers published on topic *t* authored by the set of active authors *A* during the interaction window IW. Let *a* be an active author who wrote $P_a$ papers during the IW. We define the following metrics to rank active authors and select the top and bottom 10%.

*Productivity:* the count of papers *a* has authored on topic *t* during the IW. More formally, it is the cardinality of the set $P \cap P_a$.

*Impact:* the average citation count of $P_a$ from the papers in *P*.

We argue that restricting incoming citations from *P* is a good proxy for the impact that *a* has made on that topic. The average number of citations is a better indicator of excellence than the total citation count^31^. Also, considering the average instead of the sum lowers its correlation with productivity, here measured by the *overlap coefficient*, as often the most productive authors are also the most cited ones^12^. A low correlation lets us safely disregard the confounding effects of the two metrics and allows us to treat them as fairly independent variables. Correlation statistics are reported in Supplementary Tables S4–6 online. Although citation-based measures are frequently used to quantify research impact, we are aware of the influence of social structures and other hidden biases on scholarly citation behavior^32^. Using more sophisticated measures, however, is beyond the scope of this present work.

Statistical test for difference of samples To test whether two independent samples $X_1$ and $X_2$ are different concerning their means $\mu _1$ and $\mu _2$, we assume the null hypothesis $H_0$ that their means are the same, i.e*.*, $H_0: \mu _1 = \mu _2$. Next, we compute the mean and 95% confidence interval of the distribution of the difference of their means, i.e*.*, $(\mu _1 - \mu _2)$, using bootstrapping^33^. We reject the null hypothesis $H_0$ at $p < 0.05$ if the confidence interval of $(\mu _1 - \mu _2)$ *does not* contain 0^34^. In other words, $X_1$ and $X_2$ are considered statistically different at $p < 0.05$ if the 95% confidence interval of the difference of their respective means does not contain 0. Furthermore, a positive mean of the difference indicates that $X_1 > X_2$, while a negative mean indicates $X_1 < X_2$.

In our experiments, we aggregate the differences $X_1 - X_2$ across the reference years for a given topic, and then carry out the procedure described above.

Target activation probability Let *n*(*k*) be the number of inactive authors with exactly *k* contacts during the exposure window, of whom *m*(*k*) become active in the observation window. The *target activation probability* *P*(*k*) is the probability of becoming active after having exactly *k* contacts, defined as

1 $$\begin{aligned} P(k) = \frac{m(k)}{n(k)}. \end{aligned}$$

The *cumulative target activation probability* *C*(*k*) with *k* or more contacts is given by

2 $$\begin{aligned} C(k) = \tfrac{\sum _k^\infty m(k)}{\sum _k^\infty n(k)}. \end{aligned}$$

Simple baseline for membership closure Let *p* represent the probability of activation from a single contact. The probability of activation having *k* contacts, acting independently of each other, is $P_\text {base}(k) = 1 - (1 - p)^k$. We compute *p* from the observed data using Eq. (1) as $p = P(1) = \tfrac{m(1)}{n(1)}$. This is the fraction of inactive authors with *exactly* one contact who became active as $P_\text {base}(1) = 1 - (1 - p)^1 = p$. Like before, we calculate the cumulative target activation probability for the baseline $C_{\text {base}}(k)$ with *k* or more contacts as

3 $$\begin{aligned} C_{\text {base}}(k) = \frac{\sum _k^\infty P_\text {base}(k) \cdot n(k)}{\sum _k^\infty n(k)}. \end{aligned}$$

The denominator is the same as in Eq. (1) and comes from the observed data. The numerator represents the expected number of active authors if the contacts affect the activation independently.

Source activation probability Let $n_a$ be the number of exclusive inactive coauthors of an active author *a* in the IW. Let $m_a$ be the number of those exclusive inactive coauthors who become active in the AW. The *source activation probability* of scholar *a* is thus

4 $$\begin{aligned} P_s^a=\frac{m_a}{n_a}. \end{aligned}$$

We stress that, for the probability to be well-defined, $n_a$ must be greater than zero. Therefore, in our calculations, we focused on active authors with at least one exclusive inactive coauthor.

For any $0 \le f \le 1$, we compute the fraction $C_s(f)$ of all active authors whose source activation probability is greater than or equal to *f*. $C_s(f)$ is the complementary cumulative probability distribution of the source activation probability $P_s^a$. As expected, $C_s(f)$ quickly decreases to 0 with increasing *f*. Because the curves corresponding to two sets of active authors are effectively indistinguishable at the tail, we compare a pair of points at some threshold $f^*$. We call $C_s(f^*)$ the *cumulative source activation*.

The choice of the threshold $f^*$ is important. Setting it to 0 or 1 would return the same probability for both sets of authors. It should not also be too small for numerical reasons. For example, if there are only five inactive coauthors, the smallest non-zero fraction cannot be smaller than $1/5=0.20$. Choosing too high a value instead would lead to weaker statistics. So, we fix the value at 0.10 for the results in the main text (Figs. 4 and 5), and at 0.20 in the Supplementary Figs. S3 and S4 online.

Chaperoning propensity Let $m_a$ be the number of exclusive inactive coauthors of an active author *a* who become active in the AW, which is the same as the numerator of Eq. (4). Let $i_a$ be the number of those authors who write their first paper on topic *t* with *a* in the AW. The *chaperoning probability* of *a* is defined as

5 $$\begin{aligned} P_c^a = \frac{i_a}{m_a}. \end{aligned}$$

We define the *chaperoning propensity* $P_c(f)$ corresponding to a specific threshold $f \in [0, 1]$ as the fraction of all active authors with $P_c^a \ge f$. We use the aforementioned values of 0.10 (Figs. 4 and 5) and 0.20 (Supplementary Figs. S3 and S4 online) for the threshold *f*.

### Supplementary Information

Supplementary Information.

## Data Availability

The datasets generated during and/or analyzed during the current study are available in the *Collaboration-Topic-Switches* repository on GitHub.
