# Supplementary material for: Collaboration and topic switches in science
Source: Sci Rep. 2024 Jan 13;14:1258. doi: 10.1038/s41598-024-51606-6 (PMC10787828; doi:10.1038/s41598-024-51606-6)
Supplement: Supplementary file 1 — Supplementary Information. [file 41598_2024_51606_MOESM1_ESM.pdf]

Supplementary Materials for
Collaboration and topic switches in science

Sara Venturini[+], Satyaki Sikdar[+], Francesco Rinaldi, Francesco Tudisco, and Santo Fortunato[*]

[*]Corresponding author: santo@indiana.edu
[+]these authors contributed equally to this work

This PDF file includes:

Figs. S1 to S4
Tables S1 to S6

Supplementary Table S1. Summary information for Physics topics. #Papers: average number of Papers. #Authors: average number of active authors. Averages are computed over all time windows selected for a topic.

**Topic**	**#Windows**	**Interaction Window**		**Activation Window**	
		#Papers	#Authors	#Papers	#Authors
Gravitational Wave	10	3,613.70	5,745.20	5,486.40	9,160.30
Dark Matter	13	6,433.69	8,348.23	9,203.38	12,346.00
Fluid Dynamics	16	5,290.75	11,950.38	7,231.25	16,960.50
Soliton	18	4,004.39	5,715.61	4,700.89	7,014.89
Supersymmetry	20	5,328.85	4,827.45	5,470.75	5,361.25
Statistical Physics	23	88,147.52	109,702.70	105,018.87	137,680.65
Superconductivity	23	24,038.35	33,606.04	23,218.52	34,874.74

Supplementary Table S2. Summary information for Computer Science topics. #Papers: average number of Papers. #Authors: average number of active authors. Averages are computed over all time windows selected for a topic.

**Topic**	**#Windows**	**Interaction Window**		**Activation Window**	
		#Papers	#Authors	#Papers	#Authors
Compiler	13	3,786.31	7,869.23	4,208.46	9,701.92
Mobile Computing	13	6,356.00	13,844.77	6,828.77	15,827.85
Cryptography	15	9,706.47	15,181.93	14,865.13	25,218.93
Cluster Analysis	21	18,585.57	36,645.95	30,996.52	63,910.10
Image Processing	23	13,149.65	28,191.35	16,617.65	38,089.70
Parallel Computing	23	31,453.30	48,006.87	38,271.61	61,960.22

Supplementary Table S3. Summary information for Biology & Medicine topics. #Papers: average number of Papers. #Authors: average number of active authors. Averages are computed over all time windows selected for a topic.

**Topic**	**#Windows**	**Interaction Window**		**Activation Window**	
		#Papers	#Authors	#Papers	#Authors
Protein Structure	19	6,379.95	17,583.68	7,149.11	20,967.63
Genome	23	28,066.09	71,481.87	44,089.78	114,696.48
Peptide Sequence	23	12,347.48	43,348.96	9,733.04	37,330.09
Alzheimer's Disease	23	9,313.78	22,628.30	11,723.22	31,624.61
Neurology	23	9,260.17	26,046.57	12,795.70	39,515.00
Chemotherapy	23	36,280.48	104,649.39	47,760.09	143,505.65
Radiation Therapy	23	30,926.39	76,314.48	43,963.57	110,397.96

Supplementary Table S4. Physics average Overlap Coefficient between the top and the bottom 10% of active authors selected based on Productivity and two different definitions of Impact. The first definition uses C_{avg} and is used in the main text. The second definition uses C_{tot}. The degree of overlap is significantly greater for C_{tot}.

**Topic**	**Top 10%**		**Bottom 10%**	
	C_{avg}	C_{tot}	C_{avg}	C_{tot}
Gravitational Wave	0.33	0.59	0.14	0.14
Dark Matter	0.31	0.56	0.15	0.15
Fluid Dynamics	0.24	0.38	0.11	0.11
Soliton	0.30	0.54	0.14	0.13
Supersymmetry	0.30	0.58	0.17	0.16
Statistical Physics	0.32	0.56	0.13	0.13
Superconductivity	0.26	0.60	0.16	0.15

C_{avg}: Average of incoming citations from papers on the topic.
C_{tot}: Sum of incoming citations from papers on the topic over all windows.

Supplementary Table S5. Computer Science average Overlap Coefficient between the top and the bottom 10% of active authors selected based on Productivity and two different definitions of Impact. The first definition uses C_{avg} and is used in the main text. The second definition uses C_{tot}. The degree of overlap is significantly greater for C_{tot}.

Topic	Top 10%		Bottom 10%	
	Mean	Sum	Mean	Sum
Compiler	0.27	0.46	0.12	0.11
Mobile Computing	0.25	0.41	0.12	0.12
Cryptography	0.28	0.51	0.12	0.12
Cluster Analysis	0.25	0.41	0.12	0.12
Image Processing	0.25	0.42	0.12	0.11
Parallel Computing	0.24	0.53	0.13	0.13

C_{avg}: Average of incoming citations from papers on the topic.
C_{tot}: Sum of incoming citations from papers on the topic over all windows.

Supplementary Table S6. Biology & Medicine average Overlap Coefficient between the top and the bottom 10% of active authors selected based on Productivity and two different definitions of Impact. The first definition uses C_{avg} and is used in the main text. The second definition uses C_{tot}. The degree of overlap is significantly greater for C_{tot}.

**Topic**	**Top 10%**		**Bottom 10%**	
	Mean	Sum	Mean	Sum
Protein Structure	0.22	0.46	0.13	0.13
Genome	0.22	0.50	0.13	0.13
Peptide Sequence	0.18	0.41	0.12	0.12
Alzheimer's Disease	0.19	0.55	0.13	0.14
Neurology	0.16	0.37	0.12	0.12
Chemotherapy	0.22	0.54	0.13	0.13
Radiation Therapy	0.24	0.54	0.13	0.13

C_{avg}: Average of incoming citations from papers on the topic.
C_{tot}: Sum of incoming citations from papers on the topic over all windows.

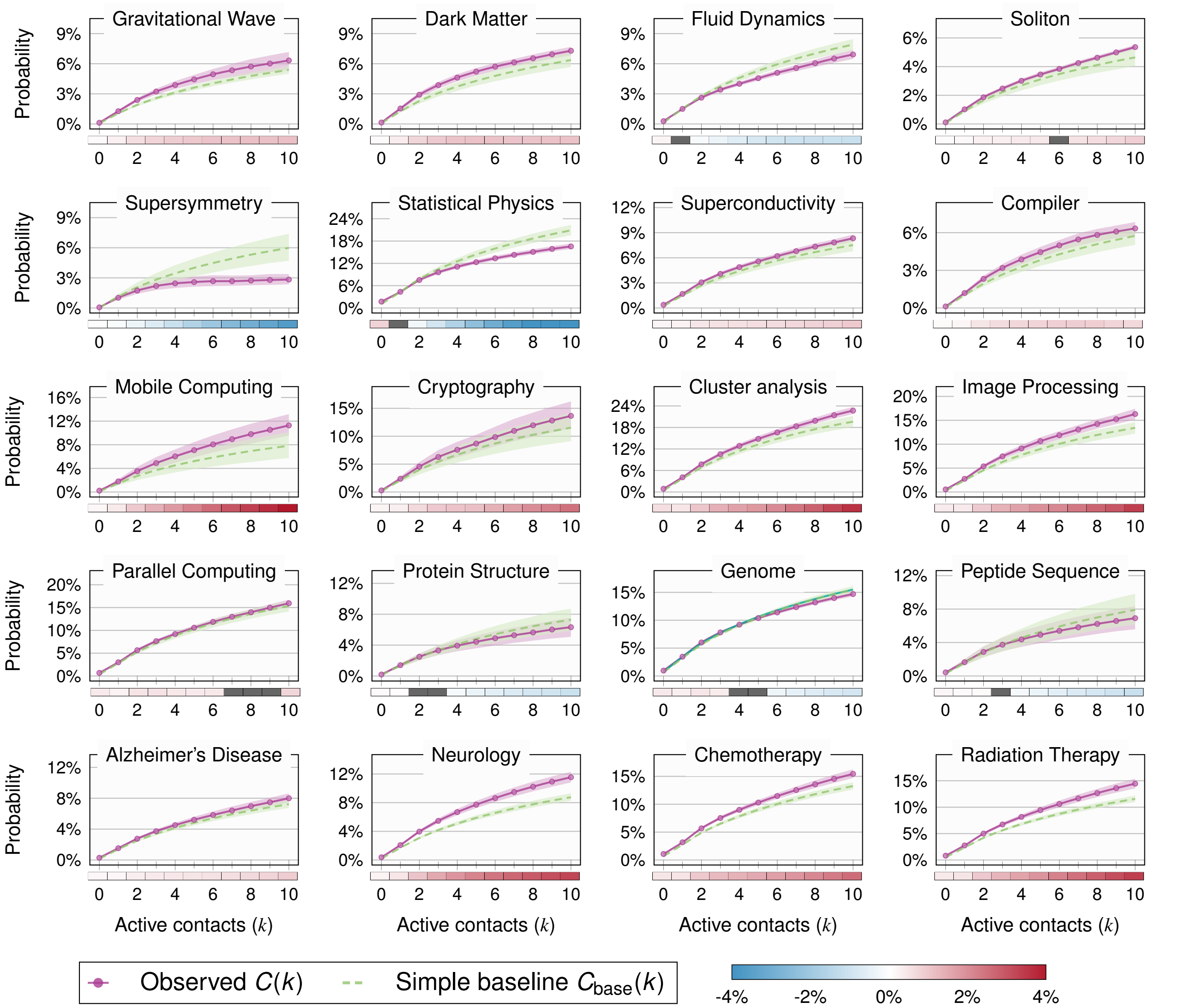

Supplementary Figure S1. Experiment I. Same as main Fig. 2, but here the number of contacts is the number of papers written with active coauthors in the IW. Cumulative target activation probability (in purple) for inactive authors in the AW with shaded 95% confidence intervals. For each k, the y-value indicates the fraction of inactive authors with at least k active contacts in the IW who became active in the AW. The dashed green line with shaded errors represents the baseline described in the text, corresponding to independent effects from the coauthors. The heatmap below the x-axis shows the mean difference between the observed and baseline curves for each k-value. It is gray if the 95% confidence interval contains 0, denoting the k-values where the points are statistically indistinguishable at p-value 0.05. Positive and negative deviations from the baseline are in red and blue, respectively.

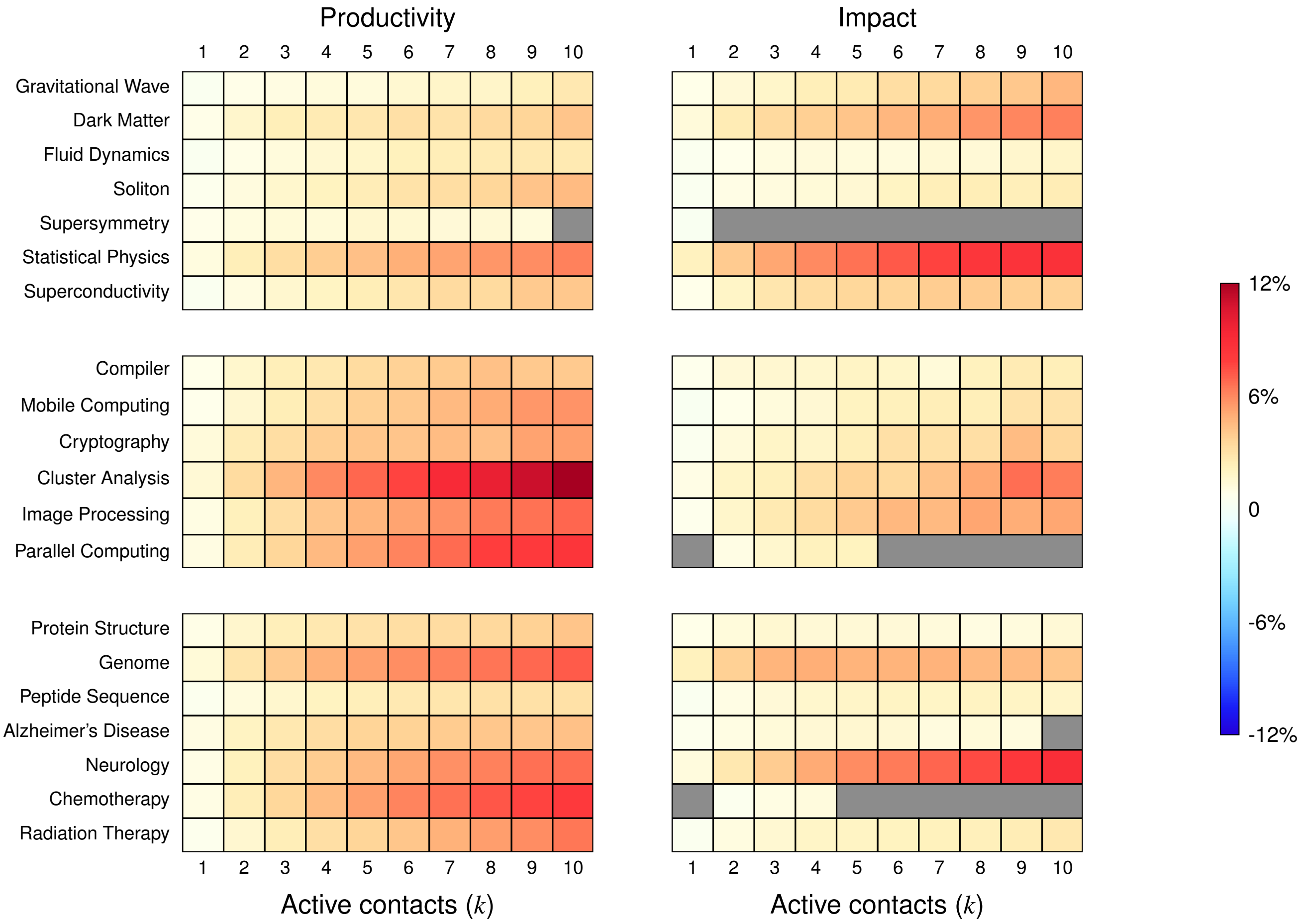

Supplementary Figure S2. Experiment I. Similar setup as main Fig. 3, but here the number of contacts is the number of papers written with active coauthors in the IW. Heatmaps showing the mean difference between the cumulative target activation probabilities of the inactive authors in the AW who had exclusive contacts with the top 10% and bottom 10% of active authors, respectively, selected according to productivity (left) and impact (right) in the IW. The cells are gray if the 95% confidence interval contains 0. The majority of red cells indicate that the cumulative target activation probabilities for contacts with the top 10% are higher than those with the bottom 10%.

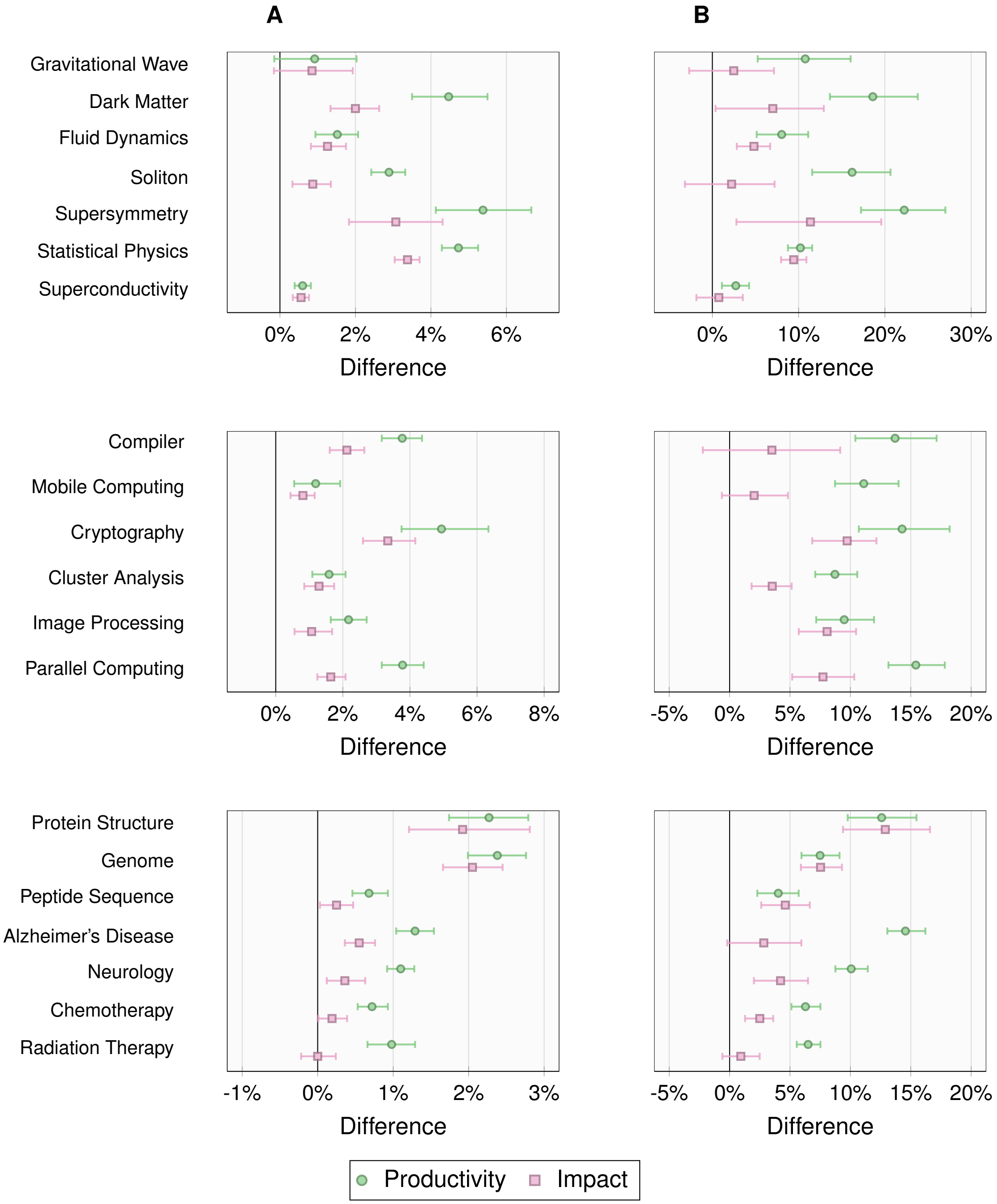

Supplementary Figure S3. Experiment II. Similar setup as main Fig. 4, with threshold $f^{\star} = 0.20$. (A) Mean and 95% confidence interval of the means of the difference between the cumulative source activations of active authors in the top 10% and bottom 10% based on productivity (green circles) and impact (pink squares). (B) Mean and 95% confidence interval of the means of the difference between the cumulative chaperoning propensities of active authors in the top 10% and bottom 10% based on productivity (green) and impact (pink). A positive difference indicates that the effect is stronger for the top 10% active authors.

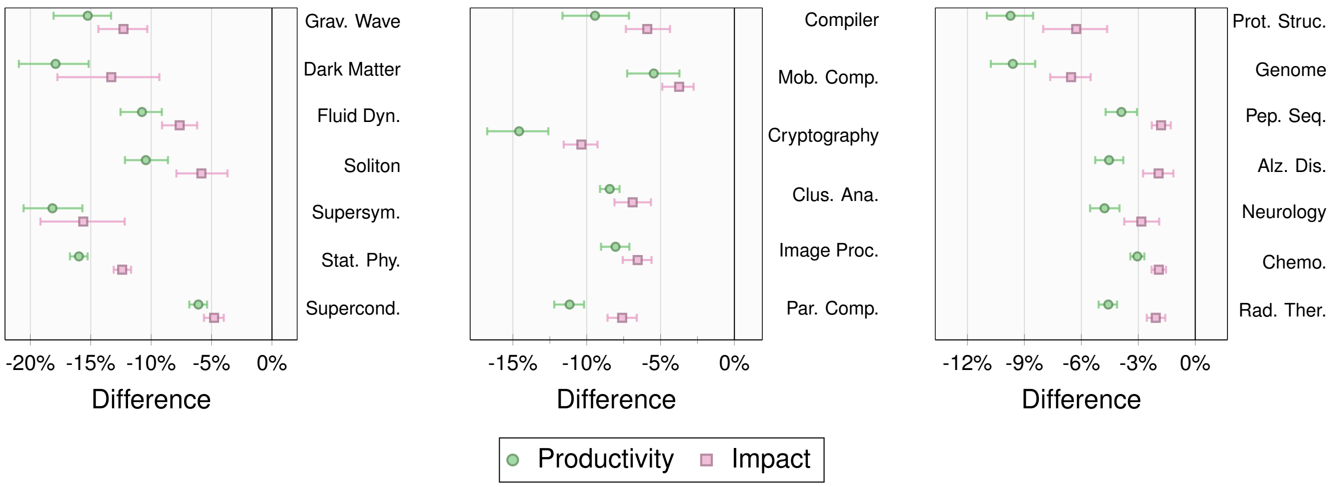

Supplementary Figure S4. Experiment II. Similar setup as main Fig. 5, with threshold $f^{\star} = 0.20$. Dilution effect. The mean and 95% confidence interval of the mean of the difference between the cumulative source activations of active authors in the top 20% and bottom 20% bins, based on the average number of coauthors, from the set of top 10% active authors in productivity (green circles) and impact (pink squares). A negative difference across the topics indicates a *dilution* effect, wherein coauthors of prominent active scholars with less collaborators (on average) are more likely to switch topics.
